# Addition of Grape Skin and Stems Extracts in Wines during the Storage to Reduce the Sulfur Dioxide: Impact on Red Wine Quality

**DOI:** 10.3390/ijerph18052783

**Published:** 2021-03-09

**Authors:** Rocío Casquete, María José Benito, Francisco Pérez-Nevado, Ana Martínez, Alberto Martín, María de Guía Córdoba

**Affiliations:** 1 School of Agricultural Engineering, University of Extremadura, Avda, Adolfo Suárez s/n, 06071 Badajoz, Spain; rociocp@unex.es (R.C.); fpen@unex.es (F.P.-N.); amartinehi@alumnos.unex.es (A.M.); amartin@unex.es (A.M.); mdeguia@unex.es (M.d.G.C.); 2 Avda. de la Investigación s/n, Campus Universitario, University of Research Institute of Agro-Food Resources (INURA), 06006 Badajoz, Spain

**Keywords:** wine, phenolic extracts, by-products, SO_2_, antimicrobial activity

## Abstract

This study aimed to evaluate the usefulness of bioactive extracts obtained from red wine by-products, such as grape skins and stems, for reducing or eliminating the use of SO_2_ in red wine production. Special attention was focused on guaranteeing the microbiological stability of the red wines and protecting them against oxidation. Therefore, the antioxidant and antimicrobial activities of the extracts and red wines were studied. Red grape stems and skins, by-products of the wine industry, from six types of monovarietal wines, were used. Extracts obtained from stems displayed higher concentrations of total phenolic compounds and higher in vitro antioxidant activity. Both stem and skin extracts demonstrated higher antimicrobial activity against pathogenic bacteria and lower activity against yeasts. In the wines produced, higher antimicrobial and antioxidant activities were observed, mainly in the skin extract batches. This study highlights that bioactive extracts obtained from by-products of wine making could be used to reduce or eliminate the use of SO_2_ in wine production. In this way, healthier red wines could be obtained while guaranteeing their microbiological stability and protecting them from oxidation. Furthermore, the use of these by-products is strongly associated with the circular economy, as they could help to reduce the environmental impact of the wine industry.

## 1. Introduction

Sulfur dioxide (SO_2_) is the most used preservative along the wine production processing chain, from pressing to bottling [1]. This compound is well known for its antimicrobial properties and antioxidant activity against non-enzymatic oxidation of wines. However, it also functions as an antioxidant to inhibit oxidase enzymes typical of grapes, such as tyrosinase or laccase, in a lower range [2,3]. The application of this compound is especially relevant in the production of white wines to avoid losses of sensorial quality due to oxidation reactions. In this sense, it has a great influence on sensorial characteristics, preventing browning and loss of color and aroma complexity [2]. Moreover, it helps to control the presence of undesirable microorganisms, such as non-*Saccharomyces* yeast or acetic or lactic acid bacteria [2,4,5].

Despite the advantages indicated above, SO_2_ is a very reactive molecule that can negatively affect wine quality, causing sensorial modifications, and can also have negative effects on the health of the consumer. Several harmful health effects of SO_2_ ingestion have been reported, mainly in asthmatics and sulphite-sensitive wine consumers, including headache, diarrhea, dermatitis, urticaria, and bronchoconstriction, among others [3,6]. In addition, SO_2_ bioaccumulation has been observed, related to the pathogenesis of lung cancer [7]. For these reasons, its use is strictly controlled by law, and it would be desirable to substitute this compound with another less harmful one.

The current trend is to search for other preservatives and technologies that can replace or reduce the use of this compound in wine, guaranteeing its microbiological stability and protection against oxidation without affecting its organoleptic properties. Among these new technologies are physical methods, such as the application of electrical pulses, ultrasound, ultraviolet radiation, or high hydrostatic pressure [8,9]. Additionally, chemical, or biological methods, such as the application of dimethyl dicarbonate, silver complexes, bacteriocins, chitosans, and lysozymes have been applied to wines [10,11,12,13]. One of the most novel substitutive techniques is the use of plant extracts or phenolic compounds, which present antimicrobial and antioxidant activities, in different kind of wines. As an alternative to SO_2_, Raposo et al. [14] elaborated Sauvignon white wines with higher antioxidant activity by using hydroxytyrosol-enriched products, one obtained by synthesis and another from olive by-products.

One of the most interesting current trends is the use of wine by-products, such as stems or grapevine shoots, grape seeds, or stems, because of their richness in antioxidant and antimicrobial compounds [15,16,17,18,19]. This not only guarantees the preservation of the wine but also allows the revaluation of by-products. Several studies have demonstrated the antimicrobial capacity of phenolic compounds from by-products in wines, showing that they had different antimicrobial effects depending on the microorganism and the type of wine. The antimicrobial activity of a pure stilbene extract obtained from vine shoots was analyzed against undesirable microorganisms in the wines [19]. This extract, rich in E-ε-viniferin and E-resveratrol, had higher activity against spoilage yeast (such as *Brettanomyces bruxellensis*, *Zygosaccharomyces bailli*, *Haseniaspora uvarum*, or *Candida zemplinina*) than against lactic acid bacteria (such as *Lactobacillus hilgardii*, *Oenococcus oeni*, or *Pediococcus pentasaceus*), which can be undesirable, especially in white and rosé wines, due to its lack of freshness or the production of different undesirable compounds. Aqueous extracts obtained from grape seeds and stems applied in white vinification, alone or in combination with colloidal silver complex, had an antimicrobial effect against lactic and acetic bacteria like that of sulfur dioxide [18]. Other studies conducted with extracts rich in phenolic compounds obtained from grape by-products, skins, seeds, and stems, were effective against pathogenic bacteria [15,17,20,21]. The antioxidant activity of extracts obtained from wine by-products was studied in different types of wines. In the studies of Marchante et al. [18], the extracts obtained from grape seeds and stems applied in white vinification did not appear to have effective antioxidant activity; only wines with stem extracts had similar scores to those elaborated with SO_2_. Studies were performed by adding commercial formulas containing plant gallic and ellagic acids extracted from grape to sparkling white wines; nevertheless, these compounds did not appear as effective as antioxidants compared with SO_2_ [16]. Esparza et al. [22] studied the antioxidant effects of a commercial vine wood extract and a grape stem extract at the fermentation stage of Tempranillo winemaking and obtained wines with organoleptic properties similar to or even better than those of wines treated with SO_2_.

However, as stated above, most of these studies do not conclusively support complete replacement of SO_2_ in wines, and further studies are needed to discover extracts that suit this purpose. The aim of this study was to evaluate the usefulness of bioactive extracts obtained from wine by-products to replace or decrease the use of SO_2_ in wine production. This would make it possible to produce healthier wines, guarantee their microbiological stability, and protect them against oxidation.

## 2. Materials and Methods

### 2.1. Plant Extracts Obtained by Ultrasound

Red grape stems and skins of Tempranillo variety, by-products of the wine industry provided by a winery located in the Extremadura Region, Spain, were used in this work. The by-products were dried in a forced ventilation stove at 45 °C for 48 h. After drying, the samples were ground and vacuum-packed in plastic bags. Finally, samples were stored at room temperature until use.

Dry samples (10 g) were mixed with 60 mL of ethanol (70% *v*/*v*). Phenolic compounds were extracted using an ultrasonic bath (360 W, J.P. Selecta, s.a. Barcelona, Spain), operating at a frequency of 50/60 Hz and power of 220 V for 1 h in the absence of light at 50 °C and filtered. This process was repeated twice. Excess ethanol was removed by heating at 37 °C in a rotary evaporator under vacuum. The resultant aqueous extracts were combined, lyophilized, packaged in vacuum bags, and stored at room temperature in a desiccator until use.

### 2.2. Total Phenolic Content and Functional Properties of Extracts

#### 2.2.1. Total Phenolic Content (TPC)

The total phenolic content (TPC) of the extracts diluted to 10 mg extract/mL with ethanol was determined using Folin-Ciocalteu reagent by the method described by Wettasinghe and Shahidi [23] in a UV-1800 spectrophotometer (Shimadzu Scientific Instruments, Columbia, MD, USA). Gallic acid was used as a standard. The results are expressed as mg gallic acid equivalent (GAE)/100 g of extract. All experiments were conducted in triplicate.

#### 2.2.2. Antioxidant Activity by Free Radical-Scavenging Ability Using a Stable DPPH Radical and ABTS Radical Cation

The antioxidant activity of the extracts diluted to 10 mg extract/mL with ethanol was determined by bleaching of the purple-colored solution of 1,1-diphenyl-2-picrylhydrazyl radical (DPPH) according to the method of Teixeira et al. [24]. Quantification was performed by plotting the values against a Trolox calibration curve. Results were expressed in mg of Trolox/100 g of extract. All experiments were conducted in triplicate.

The free radical scavenging capacity of extracts was also determined using the ABTS (2,2′-azino-bis-(3-ethylbenzothiazoline-6-sulfonic acid) radical cation decolorization as-say, according to the procedure proposed by Cano et al. [25], slightly modified (*n* = 3). The initial absorbance value at λ 730 nm was then compared with the absorbance obtained after 20 min of reaction. The results were expressed as mg of Trolox/100 g of fresh plants.

#### 2.2.3. Antimicrobial Activity

Foodborne pathogenic bacteria and altering yeasts in wine obtained from the Spanish type culture collection (CECT) were used to evaluate the antimicrobial activity. The pathogenic microorganisms were *Staphylococcus aureus* CECT 976, *Salmonella choleraesuis* CECT 4395, *Escherichia coli* CECT 4267, *Bacillus cereus* CECT 131, *Listeria monocytogenes* CECT 911 and *Listeria innocua* CECT 910. The spoilage yeasts were *Candida boidinii* CECT 11153, *Kregervanrija fluxuum* CECT12787, *Priceomyces carsonii* CECT 10230, and *Zygosacharomyces bailii* CECT 11043.

Target cell suspensions were prepared from cultures incubated overnight at 37 and 25 °C on brain-heart infusion agar (BHI; Oxoid) and yeast peptone and dextrose extract (YPD; Oxoid) agar for bacteria and yeast, respectively. After the incubation period, colonies were transferred to a sterile Peptone Water solution to obtain a turbidity equivalent to 0.5 McFarland standards. Next, 1 mL of each suspension was pipetted into separate sterile petri dishes to which 20 mL of molten BHI and YPD with 1% agar (45 °C) for the bacteria and yeast, respectively, were added. Once set, 10 μL of aqueous extracts at different concentrations (1.5, 0.7, and 0.4 mg/mL) was added. Sterile distilled water instead of active compounds was used as a negative control. The plates were incubated overnight at 37 and 25 °C for bacteria and yeast, respectively, and the diameter (mm) of the resulting zone of inhibition was measured.

### 2.3. Treatment of Wines with By-Product Extracts and Storage

The effect of by-product extracts on wine during storage was analyzed. Non-filtered red wines from of the 2019 vintage, obtained after alcoholic and malolactic fermentations, were treated with the extracts and with SO_2_. All the red wines used in this study were supplied by the same winery located in the Extremadura Region, Spain, using the same technology based on a classical process of red wine production. Six types of monovarietal wines were used in the study: Petit Verdot, Tempranillo, Cabernet Sauvignon, Cabernet Franc, Syrah, and Malbec.

SO_2_ was added as a solution of potassium metabisulphite (purity higher than 95%) in distilled water.

A total of 36 batches of 2 L of each wine was prepared, using the six monovarietal wines and six different treatments, which consisted of the addition of (i) 30 mg/L of free SO_2_, which acted as the control wine plus SO_2_ (CS); (ii) 1 g/L of lyophilized grape stem extract (R); (iii) 1 g/L of lyophilized grape stem extract plus 30 mg/L of free SO_2_ (RS); (iv) 1 g/L of lyophilized grape skin extract (H); (v) 1 g/L of lyophilized grape skin extract plus 30 mg/L of free SO_2_ (HS); (vi) control wines without SO_2_ nor extracts (C). All batches of wine were stored at room temperature for 60 days. Microbiological and physical–chemical assays were carried out at various time points (0, 15, 30, 45, and 60 days of storage). All assays were performed in triplicate.

### 2.4. Microbiological Counts

For the microbial counts, 0.1 mL aliquots of each wine sample were taken aseptically, transferred to sterile tubes, 10-fold diluted with 1% peptone water (Pronadisa, Alco-bendas, Madrid, Spain), and homogenized for 120 s using a vortex. Serial 10-fold dilutions were prepared from the same solution and inoculated onto agar plates. Plate count agar (PCA, Oxoid) was used for mesophilic aerobic bacteria counts at 30 °C for 48 h. LAB were grown in MRS agar (Oxoid) of which the pH was adjusted to 5.6 with acetic acid (10%), with incubation at 37 °C for 2 days under anaerobic conditions. Acetic acid bacteria were grown on GYC (5% glucose, 1% yeast extract, 0.5% calcic carbonate, 2% agar) plates. Plates were incubated under aerobic conditions at 30 °C for 2 days, and the yeasts count was determined on potato dextrose agar (PDA) agar at 25 °C for 2 days.

For proper counting, plates with 30 to 300 colony forming units (CFUs) were considered, with the results expressed as log CFU g^−1^.

### 2.5. Physicochemical Analysis

pH, total and volatile acidity, total and free SO_2_ and alcoholic strength were determined by official analytical methods established from common analytical methods in the wine sector [26]. The pH was measured using a Crison mod. 2002 pHmeter (Crison Instruments, Barcelona, Spain). A METROHM model 855 Robotic Titrosampler automatic titrator and a Glass Chem Kombo-2 VA-SO2 still were used to measure total and volatile acidity, respectively. The alcoholic strength of wines by volume (% vol) was determined by using densiometric measurements of a distillate obtained with a distillation unit (Selecta-Pro-Nitro S). Samples were analyzed in triplicate.

### 2.6. Antioxidant Activity

The method was based on the stability of the 1,1-diphenyl-2-picrylhydrazyl radical described in Section 2.2.2. Sample dilutions (50 μL) were added to 2.950 mL of a methanolic DPPH radical solution (Sigma-Aldrich, Tres Cantos, Madrid, Spain). Absorbance was measured after 30 min of reaction, at 515 nm in a UV–Vis spectrophotometer (Shimadzu UV spectrophotometer UV-1800), using methanol to set zero. Quantification was performed by plotting the values obtained against a Trolox calibration curve. Results were expressed in mg of Trolox per 100 mL of wine. Samples were analyzed in triplicate.

### 2.7. Statistical Analysis

Statistical analysis was performed using the SPSS statistical program for Windows (IBM Corp. 2011. SPSS Statistics, Version 25.0, Armonk, NY, USA). The mean values were studied using a two and three-way analysis of variance (ANOVA). The mean values were separated for comparison by Tukey’s honest significant difference (HSD) test (*p* ≤ 0.05). In addition, principal component analysis (PCA) of a correlation matrix of the variables was performed.

## 3. Results

The results for total phenolic content (TPC) and antioxidant activity of the stem and grape-skin extracts are presented in Table 1. The stem extracts had higher concentrations of TPC (2693.28 mg GAE/100 g) than the skin extracts (TPC: 1689.42 mg GAE/100 g). When the antioxidant activity of stem and grape-skin extracts was evaluated using two methods (DPPH and ABTS), both methods higher antioxidant activity was found in the stem extracts (421.75 and 821.76 mg Trolox/100 g, respectively) than in the skin extracts (173.81 and 490.29 mg Trolox/100 g, respectively). In stem extracts, a higher TPC was determined when compared with the grape-skin extracts, which most probably explains the higher antioxidant activity observed in the former.

Table 2 shows the antimicrobial activity results for the different extracts against the bacteria and yeasts tested. Both the stem and grape-skin extracts showed antimicrobial activity against all the bacteria tested; however, none of the extracts showed activity against *Z. bailii* and, in the case of stems, no effect against *C. boidinii* was observed. In the case of the bacteria tested, the lower concentrations did not show any effect against *L. innocua*, *L. monocytogenes* and *S. aureus*. Moreover, in the yeasts tested, the same result was observed at low concentrations of the extract.

Table 3 shows the results of the physical–chemical analysis performed on the wines used in the study. The pH values were similar for all the wines, ranging from 3.6 to 3.8. The varieties Tempranillo, Cabernet Sauvignon and Cabernet Franc showed higher total acidity values (*p* < 0.05) than the other wines. However, the values of volatile acidity in the wine Petit Verdot was significantly higher than that of the other types of wines. Regarding total and free SO_2_ levels, Petit Verdot wine presented the highest values, ranging from 60 and 30 mg/L of total and free SO_2_, respectively. The antioxidant activity of the wines was between 159 and 244 mg Trolox/100 mL, the highest values (*p* < 0.05) corresponding to Petit Verdot and the lowest to Syrah. The alcohol content was similar among all types of wines used (Table 3).

Regarding the analysis carried out on the monovarietal wines treated with the extracts and SO_2_, Table 4 shows the results of aerobic mesophilic bacteria, lactic acid bacteria, and yeast counts of the wines during the storage period. Acetic acid bacteria were not included because no growth was detected in any of the wine batches tested. The wine does not seem to be a suitable medium for the growth of these bacteria, due to characteristics such as SO_2_ content. The results in Table 4 reveal that there were no differences between wines in the growth of mesophilic aerobic bacteria. In most wines, the total mesophilic aerobic microorganisms decreased in the first few weeks until they reached a point where they remained stable and/or began to decrease, in many cases disappearing, which is a normal evolution of bacteria in an alcoholic beverage. In the Petit Verdot, Tempranillo and Cabernet Sauvignon wines, the decrease of mesophilic aerobic bacteria was significant. Among the batches, it was observed that, in general, the grape-skin and stem extracts had a significant effect on the decrease of mesophilic aerobic bacteria during the storage period, whereas the mesophilic aerobic bacteria in the control were maintained for up to 45 days and decreased at the end of storage, but the difference was not significant. With respect to lactic acid bacteria, there was a decrease in all wines and batches in general. The decrease occurred significantly earlier in batches to which grape-skin extract and grape-skin extract with sulphite were added. At the beginning of storage, there were differences (*p* < 0.05) between wine varieties in the levels of lactic acid bacteria. The decrease in these bacteria was significant in all the wines analyzed, except for Syrah and Cabernet Franc wines, where no growth of lactic acid bacteria was observed (Table 4).

Regarding the evolution of the yeast grown in the different wines and batches analyzed, a significant evolution of yeast growth was observed. In the Petit Verdot, Cabernet Sauvignon and Malbec wines, a significant effect on yeast growth was observed. However, this behavior was not observed in the rest of the wines. As for the batches, when the different extracts were applied, inoculation of wines with grape-skin extract had the greatest effect (*p* < 0.05) on yeast reduction (Table 4). No mold growth was observed since wine is not a medium that favors mold growth. Based on the results obtained, it appears that the effect on bacteria levels varies depending on the grape variety used to produce the wine. Additionally, the extracts, mainly grape-skin extract, appear to show similar antimicrobial capacity to that of SO_2_.

Table 5 shows the evolution of physicochemical characteristics of total and volatile acidity of the different wines analyzed.

Regarding total acidity, the values remained unchanged throughout 60 days of storage, although there were differences between the wine varieties. However, there were no significant differences between wines with SO_2_, and those without SO_2_.

As for the values of volatile acidity, the levels in the different wines ranged from 0.1 to 0.20 g of acetic acid per liter of wine at the beginning of storage to values of 0.27–0.44 after 60 days of storage. As can be observed, the changes in volatile acidity were similar for all wines analyzed. The values of all the batches studied, except those with skin extract, increased during the storage period (Table 5).

Therefore, the results showed that the addition of the compounds does not significantly modify these parameters of wine.

Table 6 shows the results obtained from the analysis of total and free sulfur dioxide and the antioxidant activity according to the wines and batches studied. It can be observed that the levels of total and free sulfur dioxide decreased significantly throughout wine storage, and no differences were found between the wines at the end of the storage period. However, the levels of sulfur dioxide according to the batches remained stable during storage, except for the grape-skin extract batches, which showed a significant decrease over time. Considering the results, the fact that in the present study no significant differences were observed between batches may indicate that both extracts produced an effect on the wine comparable to that of SO_2_, at least during the storage period studied.

Overall, it can be observed that the antioxidant activity decreased significantly during the first storage period and then increased. The differences found in antioxidant activity were influenced by the type of wine, with Malbec wine showing the highest levels of antioxidant activity (*p* < 0.05) (Table 6). These results showed that the antioxidant capacity of wines made with stem and skin extracts, either alone or in combination with SO_2_, was similar or superior to that of the control wine made with SO_2_.

Finally, Table 7 shows the physical–chemical analysis values of the different batches of wines at the end of the storage period. The results show that the batches with stem extract, in all types of wines, were the batches with the highest acidity values (*p* < 0.05). The levels of SO_2_, antioxidant activity, and alcohol content were similar in all the studied batches, without significant differences (Table 7). Therefore, the addition of stems and grape-skin extracts did not affect the physical–chemical parameters, as values were similar to those of the control wine.

**Figure 1:**
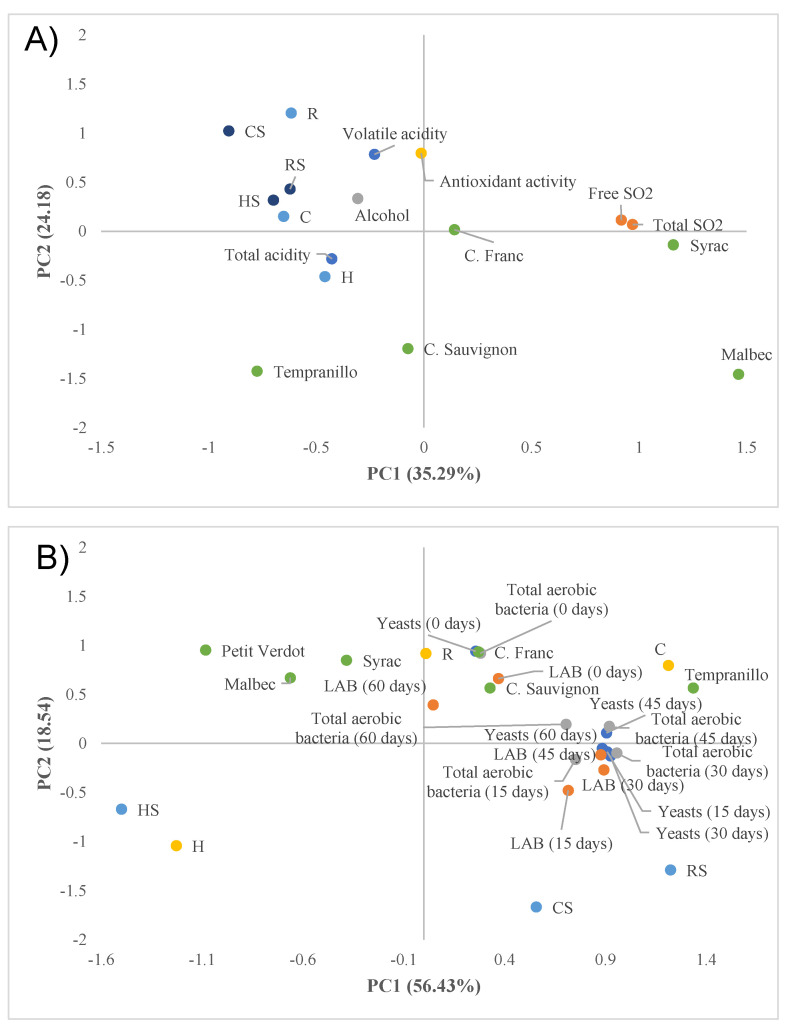
Representation of the physical–chemical variables in the samples analyzed after 60 days of storage (**A**) and microbiological analysis (**B**) in the samples analyzed during 60 days of storage in the plane defined by principal component 1 and principal component 2 of the principal component analysis. (C: control batch, CS: control batch with sulfur dioxide; R: batch of scratch, RS: batch of scratch plus sulfur dioxide, H: batch of skin and HS: batch of skin plus sulfur dioxide).

A principal component analysis was carried out with the different parameters studied, to determine the importance of these parameters in the batches and types of wine analyzed. Figure 1 shows the Figure 1 shows the two-way loadings and score plots, where principal component 2 (PC2) was plotted against principal component 1 (PC1) explaining more than 60% of total variance.

As can be observed in Figure 1A, the principal component 1 is defined by the total and free sulfur dioxide located in the positive axis which were related with the factor “wine”. As for component 2, it was defined by the variable’s antioxidant activity and volatile acidity, corresponding to 24.18% of the total variance. The highest values of antioxidant activity and acidity were obtained from the batch of scratch.

Regarding Figure 1B, when the value factors for component 1 were analyzed, the yeast counts were the ones that most influenced the variability of the different wines, with main component 1 explaining 56.43% of the total variance. As for component 2, it was defined by the control batch, with sulfur dioxide (CS) corresponding to 18.54% of the total variance.

The highest counts of mesophilic aerobic bacteria (PCA), lactic acid bacteria (MRS), and yeast (PDA) corresponded to the control batch and the Tempranillo and Cabernet (Franc and Sauvignon) wines, while the batches made with grape skins had the lowest counts, followed by the batch made from stems and the Petit Verdot and Malbec wines.

## 4. Discussion

In our studies the stem extracts had higher concentrations of TPC than the skin extracts. Makris et al. [27] studied the TPC of stem, skins, and seeds of red grape varieties and obtained higher concentrations of TPC in stems than in skins, in agreement with our results. However, other authors observed that the TPC of seeds was the highest in comparison with the scrape and skin [17,27,28]. The phenolic content of the grapes depends mainly on the variety [29]. The values of TPC reveal that by-products of the wine industry are sources of polyphenols.

Stem extracts showed higher antioxidant activity than grape-skin extracts. These results agree with previous studies that evaluated the antioxidant capacity and TPC of several grape varieties and reported a high correlation between these parameters, pointing to the fact that the antioxidant activity of wines is mainly due to its phenolic compounds [30,31]. Doshi et al. [32] investigated the antioxidant capacity of the skins, seeds, and stem extracts of two grape varieties and found that in Merlot extracts, the stems presented a higher antioxidant activity than the skins. In addition, the stem and grape-skin extracts were shown to be active against the pathogenic bacteria studied and, to a lesser extent, against yeasts, this activity is associated to phenolic compounds (Table S1). Different studies carried out with extracts rich in phenolic compounds obtained from by-products of the grape, skins, seeds and stems, have shown efficacy against pathogenic bacteria. Papadopoulou et al. [20] proved antimicrobial activity of wine extracts against *S. aureus* and less effective against *E. coli*. Katalinić et al. [15] confirmed very promising results regarding the antimicrobial activity of grape skin extracts of white grape cultivars, especially against gram-negative bacteria, such as *Salmonella*. However, Silva et al. [17] did not observe antibacterial activity in grape skin extracts against the gram-negative bacteria *Salmonella enteritidis*, *Escherichia coli* and *Pseudomonas aeruginosa*. Fewer studies have been performed regarding the impact on yeasts, but Papadopoulou et al. [20] have shown a moderate impact of wine extracts on *C. albicans*.

Based on the results obtained from the analysis of aerobic mesophilic bacteria, lactic acid bacteria and yeast behavior in different wines, it appears that the effect on these varies depending on the grape variety used to manufacture the wine and, on the extract, used, the extract obtained from the skin being the main one that presented an antimicrobial capacity similar to that of SO_2_. Gutiérrez-Escobar et al. [19] also showed that the antimicrobial capacity of the phenolic compounds in wine by-products had different antimicrobial effects depending on the microorganism and type of wine. Stilbene extract obtained from vine shoots had higher activity against yeasts than against lactic acid bacteria. On the other hand, Marchante et al. [18] found an antimicrobial effect against lactic and acetic acid bacteria from grape extracts applied in white wine processing.

Regarding the evolution of physicochemical characteristics of total acidity of the different wines analyzed, in general it was found that the differences in these parameters were only due to the type of wine and the evolution during the process, and in no case to the addition of the extracts. Marchante et al. [18] neither observed differences in total nor volatile acidity in white wines with added extracts. In the present study, no significant differences were observed between the different batches regarding the analysis of total and free sulfur, indicating that both extracts exerted an action comparable to that of SO_2_ in the wine. However, the antioxidant capacity of wines made with stems and skins extracts, either alone or in combination with SO_2_, was similar or superior to that of the control wine in accordance with the results observed by other authors [18,33]. Esparza et al. [22] found significant differences in the antioxidant activity between treatments with and without extracts (vine wood and stems) throughout the winemaking process; however, after one year of storage in bottles, no statistically significant differences from control wines were found. On the contrary, studies adding grape extracts to sparkling white wines [16] showed that these extracts appeared less effective as antioxidants than SO_2_.

## 5. Conclusions

In order to use alternative additives, such as natural extracts, in winemaking, it is necessary to investigate the antioxidant and antimicrobial activities of the extracts in vitro and in vivo, as well as the influence on the physical–chemical characteristics of the wines.

The stem extracts obtained showed higher concentrations of phenolic compounds and greater antioxidant activity in vitro. However, both stems and skin extracts presented antimicrobial activity against pathogenic bacteria to a greater extent and against yeasts to a lesser extent. In the elaborated wines, no significant differences were observed in the physical–chemical parameters due to the addition of the extracts; however, greater antimicrobial and antioxidant activity was observed mainly in the batches with skin extracts.

This study highlights that the phenolic compounds obtained from wine by-products, stems and skins could be used to replace or decrease the use of SO_2_ for wine production. In this way, healthier wines could be obtained, as well as guaranteeing the microbiological stability of the wines and protecting them from oxidation. Moreover, the use of these by-products is closely related to the circular economy, as it would contribute to reduce the environmental impact of wine industry. To complete this work, further studies should be performed in other types of wines and under different winemaking conditions as well as sensory analyses.

## Figures and Tables

**Table 1:** Total phenolic content (TPC) expressed in mg gallic acid equivalent (GAE)/100 g extract and antioxidant activity (DPPH and ABTS) expressed in mg Trolox/100 g extract of the stem and grape-skin.

Samples	TPC			DPPH			ABTS		
	Mean		SD ^1^	Mean		SD	Mean		SD
Stem	2693.28	±	169.84	421.75	±	43.98	821.76	±	4.24
Grape-skin	1689.42	±	24.39	173.81	±	31.25	490.29	±	5.61
*p* values ^2^	0.013			0.023			0.000		

^1^ SD: standard deviation. ^2^ *p* values of the variable samples are significantly different (*p* < 0.05) between extracts.

**Table 2:** Diameter of the zones of inhibition in millimeters of stem and grape-skin extract with different concentrations tested against bacteria and yeast.

		Bacteria						Yeast			
Samples	Extract (mg/mL)	1	2	3	4	5	6	7	8	9	10
**Stem**	6	11 ^ab^_1_	11 ^ab^	10 ^b^_1_	12 ^a^_1_	8 ^c^_1_	11 ^ab^_1_	-	-	10 ^b^_1_	12 ^a^
	3	7 ^b^_2_	-	8 ^b^_2_	8 ^b^_2_	7 ^b^_12_	7 ^b^_2_	-	-	10 ^a^_1_	-
	1.5	7 ^ab^_2_	-	7 ^ab^_2_	7 ^ab^_23_	6 ^b^_2_	6 ^b^_23_	-	-	8 ^a^_2_	-
	0.8	6 ^ab^_2_	-	6 ^ab^_23_	6 ^ab^_3_	6 ^ab^_2_	5 ^b^_3_	-	-	7 ^a^_2_	-
	0.4	-	-	5 ^a^_23_	-	4 ^a^_3_	-	-	-	-	-
**Grape skin**	6	10 ^e^_1_	7 ^f^_1_	28 ^a^_1_	12 ^d^_1_	10 ^e^_1_	20 ^b^_1_	10 ^e^_1_	-	18 ^c^_1_	10 ^e^_1_
	3	8 ^d^_2_	5 ^e^_1_	28 ^a^_1_	7 ^d^_2_	7 ^d^_2_	15 ^b^_2_	7 ^d^_2_	-	10 ^c^_2_	10 ^c^_1_
	1.5	6 ^d^_2_	-	28 ^a^_1_	7 ^d^_2_	7 ^d^_2_	15 ^b^_2_	6 ^d^_2_	-	10 ^c^_2_	6 ^d^_2_
	0.8	-	-	8 ^b^_2_	6 ^c^_2_	5 ^c^_3_	9 ^a^_3_	6 ^c^_2_	-	10 ^a^_2_	6 ^c^_2_
	0.4	-	-	8 ^a^_2_	-	5 ^b^_3_	9 ^a^_3_	-	-	-	-

1: *L. innocua*; 2: *L. monocytogenes*; 3: *B. cereus*; 4: *S. aureus*; 5: *S. choleraesuis*; 6: *E. coli*; 7: *C. boidinii*; 8: *Z. bailii*; 9: *K. fluxuum*; 10: *P. carsonii*; no inhibition; ^a–f^: Values with different superscripts are significantly different between microorganisms within concentrations _1–3_: Values with different subscripts are significantly different between concentrations in one microorganism.

**Table 3:** Physicochemical parameters of the types of wines used in the study.

Parameters	Petit Verdot	Tempranillo	C. Sauvignon	C. Franc	Syrah	Malbec
pH	3.6	3.7	3.7	3.6	3.8	3.7
Total acidity (g tartaric acid/L)	5.63 ^b^	6.80 ^a^	6.79 ^a^	6.18 ^ab^	5.07 ^bc^	4.58 ^c^
Volatile acidity (g acetic acid/L)	0.27 ^a^	0.12 ^b^	0.06 ^c^	0.06 ^c^	0.15 ^b^	0.15 ^b^
Total SO_2_ (mg/L)	61.70 ^a^	18.43 ^c^	17.31 ^c^	19.23 ^c^	47.91 ^b^	44.55 ^b^
Free SO_2_ (mg/L)	33.84 ^a^	12.69 ^b^	7.82 ^c^	14.49 ^b^	30.38 ^a^	29.49 ^a^
Antioxidant activity (mg Trolox/100 mL)	244.5 ^a^	180.5 ^b^	221.0 ^a^	208.4 ^ab^	159.6	237.1 ^a^
Alcohol (%)	12.42	12.46	12.83	12.5	12.41	12.83

^a–c^ Mean values with different numbers indicate statistically significant differences (*p* < 0.05) between wines.

**Table 4:** Evolution of total aerobic bacteria (log CFU/g) in PCA medium, LAB (log CFU/g) in MRS medium and yeasts (log CFU/g) in PDA medium during monovarietal wine storage (0, 15, 30, 45 and 60 days). (C: control batch, CS: control batch with sulfur dioxide; R: batch of scratch, RS: batch of scratch plus sulfur dioxide, H: batch of grape skin and HS: batch of grape skin plus sulfur dioxide).

	PCA (CFU/mL)					MRS (CFU/mL)					PDA (CFU/mL)				
	0	15	30	45	60	0	15	30	45	60	0	15	30	45	60
*Type of wine*															
Petit Verdot	6.29 ^a^	5.61 ^ab^	3.79 ^ab^	1.86 ^bc^	1.12 ^c^	5.35 ^a^_1_	4.85 ^a^_1_	2.93 ^ab^	1.46 ^b^	0.00 ^b^	5.08 ^a^	5.77 ^ab^_1_	2.85 ^ab^	1.36 ^ab^	1.07 ^b^
Tempranillo	4.62 ^a^	3.53 ^ab^	3.12 ^ab^	2.64 ^ab^	0.32 ^b^	4.36 ^a^_1_	3.19 ^ab^_12_	2.04 ^ab^	1.47 ^ab^	0.00 ^b^	4.68	2.30 _12_	2.63	1.83	1.59
Cabernet Sauvignon	5.47 ^a^	5.26 ^a^	3.52 ^ab^	2.18 ^ab^	0.60 ^b^	5.46 ^a^_1_	1.90 ^b^_12_	1.14 ^b^	1.02 ^b^	0.00 ^b^	5.48 ^a^	3.75 ^ab^_12_	3.26 ^ab^	1.78 ^ab^	0.58 ^b^
Cabernet Franc	3.95	1.46	1.57	1.57	1.21	0.00 _2_	0.00 _2_	1.37	1.43	1.40	3.59	1.16 _2_	1.12	1.85	1.13
Syrah	5.21	4.52	1.57	1.33	1.26	0.00 _2_	0.00 _2_	0.00	0.04	0.00	3.85	1.23 _2_	1.20	0.96	0.63
Malbec	4.30	2.72	1.03	0.92	0.00	3.95 ^a^_1_	0.00 ^b^_2_	0.00^b^	0.00 ^b^	0.00 ^b^	4.00 ^a^	1.25 ^ab^_2_	1.03 ^ab^	1.17 ^ab^	0.00 ^b^
*Batches*															
C	5.35	4.49	4.22	3.27	1.23	4.92	1.33	1.69	1.50	0.00	4.50	4.50	3.46	1.95	1.23
CS	5.35 ^a^	5.35 ^a^	3.91 ^ab^	1.73 ^ab^	0.00 ^b^	4.92 ^a^	4.92 ^a^	2.66 ^ab^	1.27 ^ab^	0.00^b^	4.50	4.57	3.89	1.77	1.24
R	5.35 ^a^	4.5 ^ab^	2.13 ^ab^	1.83 ^ab^	0.81^b^	4.92	2.51	2.07	1.46	0.00	4.50	2.63	2.27	2.01	1.77
RS	5.35	5.47	4.66	3.25	2.48	4.92 ^a^	3.63 ^ab^	2.49 ^ab^	1.88 ^ab^	0.00 ^b^	4.50	4.57	3.70	2.84	2.32
H	5.35 ^a^	3.00 ^ab^	1.39 ^ab^	0.77 ^ab^	0.00 ^b^	4.92 ^a^	1.15 ^ab^	0.45 ^b^	0.48 ^b^	0.00 ^b^	4.50 ^a^	1.50 ^ab^	0.44 ^b^	0.45 ^b^	0.84 ^b^
HS	5.35 ^a^	3.13 ^ab^	0.99 ^b^	0.49 ^b^	0.00 ^b^	4.92 ^a^	1.35 ^ab^	0.00 ^b^	0.33 ^b^	0.00 ^b^	4.50 ^a^	1.57 ^ab^	1.23 ^ab^	0.49 ^b^	0.00 ^b^

^a–c^ Mean values with different letters indicate statistically significant differences (*p* < 0.05) throughout storage. _1,2_ Mean values with different number indicate statistically significant differences (*p* < 0.05) between wines or batches.

**Table 5:** Evolution of total acidity (g tartaric acid/L) and volatile acidity (g acetic acid/L) during wine storage (C: control batch, CS: control batch with sulfur dioxide; R: batch of scratch, RS: batch of scratch plus sulfur dioxide, H: batch of skin and HS: batch of skin plus sulfur dioxide).

	Total Acidity (g Tartaric Acid/L)					Volatile Acidity (g Acetic Acid/L)				
	0	15	30	45	60	0	15	30	45	60
*Type of wine*										
Petit Verdot	7.18 _1_	7.85 _1_	7.65 _1_	7.54 _1_	6.77 _1_	0.16	0.22	0.17	0.26	0.43
Tempranillo	7.07 _12_	7.64 _1_	6.96 _12_	7.35 _12_	6.47 _12_	0.13	0.22	0.22	0.28	0.44
Cabernet Sauvignon	6.01 _12_	6.04 _12_	5.63 _23_	6.35 _3_	5.60 _12_	0.10	0.20	0.21	0.39	0.36
Cabernet Franc	5.06 _12_	5.56 _2_	5.13 _23_	5.64 _3_	4.84 _12_	0.20	0.30	0.24	0.45	0.42
Syrah	4.77 _2_	5.14 _2_	4.89 _3_	6.51 _23_	4.56 _2_	0.16	0.22	0.18	0.38	0.29
Malbec	6.04 _12_	5.51 _2_	5.87 _123_	5.16 _3_	4.87 _12_	0.37	0.26	0.45	0.42	0.47
*Batches*										
C	5.84	5.90	5.92	6.20	5.20	0.14 ^b^	0.19 ^ab^	0.17 ^ab^_12_	0.32 ^ab^_123_	0.39 ^a^_12_
CS	6.43	6.74	6.07	6.80	5.67	0.11 ^b^	0.14 ^b^	0.15 ^b^_12_	0.26 ^b^_23_	0.55 ^a^_1_
R	6.43	7.05	6.72	7.80	6.11	0.23 ^b^	0.35 ^b^	0.39 ^b^_1_	0.51 ^a^_1_	0.56 ^a^_1_
RS	7.57	7.86	7.61	7.13	7.35	0.19 ^b^	0.30 ^ab^	0.27 ^ab^_12_	0.46 ^a^_12_	0.40 ^ab^_12_
H	5.71	5.99	5.61	6.31	5.19	0.18	0.19	0.19 _12_	0.25 _23_	0.25 _2_
HS	6.44	6.80	6.21	7.26	5.95	0.12	0.15	0.13_2_	0.21 _3_	0.28 _2_

^a,b^ Mean values with different letters indicate statistically significant differences (*p* < 0.05) throughout storage. _1–3_ Mean values with different number indicate statistically significant differences (*p* < 0.05) between wines or batches.

**Table 6:** Evolution of SO_2_ total and free content (mg/L) and of antioxidant activity (mg Trolox/100 mL) during wine storage. (C: control batch, CS: control batch with sulfur dioxide; R: batch of scratch, RS: batch of scratch plus sulfur dioxide, H: batch of skin and HS: batch of skin plus sulfur dioxide).

	Total SO_2_ (mg/L)					Free SO_2_ (mg/L)					Antioxidant Activity (mg Trolox/100 mL)				
	0	15	30	45	60	0	15	30	45	60	0	15	30	45	60
*Type of wine*															
Petit Verdot	23.3 ^a^_3_	15.2 ^bc^_3_	18.6 ^ab^	15.5 ^bc^	11.9 ^c^	30.0	11.23	10.51	11.29	10.38	261.5 ^a^_3_	188.4 ^ab^	180.9 ^b^_2_	187.5 ^ab^_2_	199.2 ^ab^_3_
Tempranillo	21.3 ^a^_3_	16.2 ^ab^_3_	19.9 ^ab^	17.5 ^ab^	14.0 ^b^	30.0 ^a^	12.3 ^b^_3_	11.9 ^b^	12.2 ^b^	10.8 ^b^	287.1 ^a^_2_	212.5 ^b^	195.4 ^b^_2_	219.4 ^ab^_2_	227.2 ^ab^_3_
Cabernet Sauvignon	23.5 ^a^_3_	17.5 ^ab^_3_	20.3 ^ab^	19.4 ^ab^	16.8 ^b^	30.0 ^a^	12.2 ^b^_3_	12.34	12.5 ^b^	10.62	296.4 ^a^_1_	220.9 ^bc^	192.4 ^c^_2_	214.6 ^cb^_2_	275.3 ^ab^_2_
Cabernet Franc	45.3 ^a^_2_	25.9 ^ab^_2_	21.8 ^bc^	20.3 ^bc^	15.6 ^c^	30.4 ^a^	13.5 ^b^_3_	10.9 ^bc^	10.2 ^c^	9.61 ^c^	261.9 ^a^_3_	223.3 ^ab^	251.3 ^ab^_1_	183.9 ^b^_2_	226.5 ^ab^_2_
Syrah	44.9 ^a^_2_	25.9 ^b^_2_	20.1 ^c^	19.9 ^c^	15.3 ^c^	30.0 ^a^	14.8 ^b^_2_	12.7 ^bc^	10.3 ^cd^	9.78 ^d^	237.1 _5_	168.16	232.5 _2_	184.1_2_	191.2 _3_
Malbec	57.6 ^a^_1_	43.2 ^b^_1_	20.6 ^c^	19.1 ^c^	17.9 ^c^	30.0 ^a^	23.6 ^b^_1_	13.0 ^bc^	11.6 ^cd^	9.56 ^d^	245.4 ^ab^_4_	206.7 ^c^	239.6 ^b^_2_	301.5 ^ab^_1_	311.4 ^a^_1_
*Batches*															
C	34.8 _2_	25.1	19.9	18.2	15.1	30.0	15.0	11.6	10.8	9.8	246.9 ^a^	190.4 ^b^	198.3^b^	214.7 ^a^	231.7 ^a^
CS	23.8 _3_	15.5	17.5	16.5	13.6	30.0	12.0	11.7	11.6	10.6	279.9	194.4	202.9	204.4	226.7
R	36.3 _1_	26.2	22.5	19.7	16.9	30.0	14.9	12.6	12.1	10.2	278.4	216.0	216.3	217.3	242.9
RS	26.7 _3_	18.6	22.5	18.5	15.1	30.0	13.5	11.6	12.2	11.6	309.8	224.2	217.5	204.7	245.9
H	34.7 ^a^_2_	20.3 ^ab^	18.5 ^ab^	17.9 ^ab^	13.8 ^b^	30.0	13.2	11.5	11.0	10.0	258.1 ^a^	199.2 ^b^	220.8 ^a^	215.2 ^a^	228.8 ^a^
HS	21.9 _3_	15.4	18.48	17.4	13.8	30.0	11.4	11.6	12.4	10.0	277.8 ^a^	211.7 ^a^	169.6 ^b^	209.1 ^a^	252.8 ^a^

^a–d^ Mean values with different letters indicate statistically significant differences (*p* < 0.05) throughout storage. _1–4_ Mean values with different number indicate statistically significant differences (*p* < 0.05) between wines or batches.

**Table 7:** Physicochemical parameters performed on batches of various wines at the end of the storage period (C: control batch, CS: control batch with sulfur dioxide; R: batch of scratch, RS: batch of scratch plus sulfur dioxide, H: batch of skin and HS: batch of skin plus sulfur dioxide).

Batches	Total Acidity	Volatile Acidity	Total SO_2_	Free SO_2_	Antioxidant Activity	Alcohol
*Petit Verdot*						
C	4.76 ^a^	0.45 ^a^	18.56	8.56 ^a^	321.67	13.00
R	5.32 ^b^	0.60 ^b^	19.43	10.34 ^b^	291.89	12.50
H	4.54 ^a^	0.36 ^a^	15.79	9.78 ^ab^	320.567	13.00
*Tempranillo*						
C	6.25 ^a^	0.33 ^a^	11.22	8.21 ^a^	191.26	13.00
CS	5.95 ^a^	0.60 ^b^	11.06	10.00 ^b^	179.93	13.00
R	7.73 ^b^	0.63 ^b^	14.10	12.56 ^b^	218.40	13.00
RS	7.94 ^b^	0.45 ^a^	12.82	11.89 ^b^	213.66	12.50
H	6.53 ^a^	0.21 ^a^	10.58	10.90 ^b^	168.86	13.00
HS	6.25 ^a^	0.36 ^a^	11.70	8.72 ^a^	223.14	13.00
*C. Sauvignon*						
C	6.22 ^a^	0.54 ^b^	13.46	10.64	242.38	12.50
CS	5.94 ^a^	0.6 ^b^	13.14	11.67	231.58	12.50
R	6.74 ^ab^	0.63 ^b^	15.38	9.87	224.99	12.50
RS	7.31 ^b^	0.36 ^a^	14.10	11.67	243.70	12.50
H	6.28 ^a^	0.18 ^a^	13.94	9.74	239.22	12.30
HS	6.34 ^a^	0.30 ^a^	14.42	11.41	181.51	12.50
*C. Franc*						
C	5.04 ^a^	0.42 ^ab^	16.35	11.15	239.75 ^a^	13.00
CS	5.13 ^a^	0.45 ^ab^	16.67	10.13	268.47 ^bc^	13.00
R	6.24 ^b^	0.51^b^	18.75	11.15	260.30 ^bc^	12.5.0
RS	6.79 ^b^	0.39 ^a^	18.43	11.41	280.59 ^bc^	13.00
H	5.12 ^a^	0.21 ^a^	15.22	9.87	248.71 ^bc^	13.00
HS	5.27 ^a^	0.18 ^a^	15.38	10.00	353.85 ^c^	12.50
*Syrah*						
C	4.59 ^a^	0.39 ^a^	14.9 ^a^	11.28 ^a^	206.28	12.50
R	5.43 ^b^	0.60 ^b^	18.11 ^b^	9.10 ^ab^	259.25	12.50
H	4.51 ^a^	0.27 ^a^	13.78 ^a^	8.46 ^b^	213.92	12.50
*Malbec*						
C	4.31 ^a^	0.21	16.35	8.97 ^a^	188.89	12.50
R	5.22 ^b^	0.39	16.02	8.59 ^a^	202.85	12.50
H	4.14 ^a^	0.27	13.78	11.79 ^b^	181.77	12.25

^a–c^ Mean values with different numbers indicate statistically significant differences (*p* < 0.05) between batches within the same wine.

## Data Availability

Not applicable.

## Supplementary Materials

The following are available online at https://www.mdpi.com/1660-4601/18/5/2783/s1, Table S1: Tentatively identification, retention times, characteristic ions, and abundance (Arbitrary area units) of the majority compounds from stem and grape-skin extract analyzed by HPLC-ESI-QTOF.
