# Supplementary material for: Addition of Grape Skin and Stems Extracts in Wines during the Storage to Reduce the Sulfur Dioxide: Impact on Red Wine Quality
Source: Int J Environ Res Public Health. 2021 Mar 9;18(5):2783. doi: 10.3390/ijerph18052783 (PMC7967275; doi:10.3390/ijerph18052783)
Supplement: Supplementary file 1 [file ijerph-18-02783-s001.pdf]

Addition of grape skin and stems extracts in wines during the storage to reduce the Sulphur dioxide: impact on red wine quality

Rocío Casquete; María José Benito*; Francisco Pérez-Nevado; Ana Martínez; Alberto Martín; María de Guía Córdoba

SUPPLEMENTAL MATERIAL

This article contains the methodology for analysis by HPLC-ES-QTOF of SFE extract compounds and 1 supplementary table.

METHODOLOGY FOR HPLC-ES-QTOF ANALYSIS

INSTRUMENT: LC-ESI-QTOF (Agilent HPLC-QTOF Model G6530, Agilent Technologies, Palo Alto, CA, USA).

MASS_SPECTROMETRY: Ion mode negative; Collision energy 10-40 eV (N_2); Scanning m/z 100-1700.

CHROMATOGRAPHY: Column C18 (4.6 x 1.50 mm, x 4.8 µm), Agilent Technologies; Temperature 30ºC; Solvents: water with 0.1% formic acid (A) and methanol with 0.1% formic acid (B). Gradient: hold 5 min at 95A/5B, linear from 95A/5B to 10A/90B 10 min, hold 5 min at 10A/90B, linear from 10A/90B to 95A/5B 5 min, reequilibration 95A/5B (5 min). Flow rate: 0.35 mL/min.

Table S1. Tentatively identification, retention times, characteristic ions, and abundance (Arbitrary area units) of the majority compounds from stem and grape-skin extract analyzed by HPLC-ESI-QTOF.

Proposed compound	RT[1]	[M-H]-	MS/MS ions	Stem	Grape-skin
**Phenolic compounds**					
Derivate feliric acid	3,01	311	**103;**193; 149	6386957	
Sinapyl aldehyde	3,04	**149**			146843
Dihydroxycoumarin	3,15	**133**			71786
1,3-Dicaffeoylquinic acid	3,17	353	**179**; 191	20130	
Galic acid hexoside	10,94	331	**125**; 169,01	1153575	
Protocatechuic acid hexoside	11,314	315	**108,01**; 109; 152,01	602744	
Derivate syringic acid	12,192	197,01	122,99; **138,01**; 166,97; 182	102363	
Proanthocyanidin *dimer*	12,53	577,07	**289,07**; 245,08; 407,07; 425; 451	184667	42245
Malvidin-3-O-glucoside	12,64	491,17	**329,06**; 313; 314; 330	37770	23172
Proanthocyanidin *dimer*	12,82	577,07	**289,07**; 407,07;	31895	15796
Proanthocyanidin *dimer*	13,037	425	**289,07**; 245,08; 109,03; 577	534888	107290
Malvidin-acetil-hexoside	13,19	533,12	**329**; 314; 299;	96943	4286
Proanthocyanidin *trimer*	13,22	867,19	**865,19;** 866,19;		56504
Syringic acid	13,23	**197,04**	153,02	20786	
Proanthocyanidin tr*imer*	13,29	695	**289;** 407; 451; 577	50201	16901
Vanillin	13,33	**151**		54498	
(+)-Catechin	13,39	**289,07**	245,08; 125,02	294047	24935
Proanthocyanidin *dimer*	13,42	577	**407**	117662	77629
Rutin	13,518	**609,14**	300,03	90261	
Quercetin-3-O-galactoside	13,783	**463,08**	300,01	910189	
Quercetin-3-O-glucuronide	13,883	**477,08**	301,04;151	2149033	24449
Syringetin-3-O-glucoside	14,081	**507,06**	344,01;447,04	321049	

Quercitina	15,573	**301,03**	151	843172	
**Terpenoids**					
Ursolic acid	16,427	457,35	**455,35**; 456,35		25082072
**Fatty acids and derivates**					
FA 18:1 +3O	16,14	**329,22**	139; 171; 211,13	146304	
13-HOTrE®(13S-hydroxy-6Z,9Z,11E-octadecatrienoic acid)	18,05	**221,15**	177,09; 220,15; 192	42966	
Linoleic acid	18,68	280,23	**279,23**		91957554
13-HOTrE/13-KODE	19,48	**293**	275,3	47374	
Stearic acid	19,67	284,26	**283,26**		8706431
9-HODE (9S-hydroxy-10E,12Z-octadecadienoic acid)	20,225	**295,22**	171,09; 277,21	184443	
Heptadecanoic acid	20,8	**267,19**		66406	509636
Palmitic acid	23,09	**255,23**	254		17992020
Oleic acid	24,07	**281,25**	282,25		11740911
**Unknown**					
Unknown 1	14,57	**549**	345; 344; 301	37168	
Unknown 2	20,76	**469**	423	329642	
Unknown 3	20,7	270,21	**269,2**		505044

[1]RT: Retention time (min)
[3]Majority ion
